# Assessment of Still and Moving Images in the Diagnosis of Gastric Lesions Using Magnifying Narrow-Band Imaging in a Prospective Multicenter Trial

**DOI:** 10.1371/journal.pone.0100857

**Published:** 2014-07-02

**Authors:** Tomoyuki Hayashi, Hisashi Doyama, Yukihiro Shirota, Hirokazu Tsuji, Youhei Marukawa, Hajime Ohta, Kazuhiro Miwa, Takaharu Masunaga, Shuichi Terasaki, Yutaka Matano, Kunihiro Tsuji, Yoshibumi Kaneko, Toshihide Okada, Hiroshi Kurumaya, Shuichi Kaneko

**Affiliations:** 1 Department of Gastroenterology, Ishikawa Prefectural Central Hospital, Kanazawa, Ishikawa, Japan; 2 Department of Gastroenterology, Saiseikai Kanazawa Hospital, Kanazawa, Ishikawa, Japan; 3 Department of Gastroenterology, Kanazawa Municipal Hospital, Kanazawa, Ishikawa, Japan; 4 Department of Gastroenterology, Kanazawa Medical Center, Kanazawa, Ishikawa, Japan; 5 Department of Gastroenterology, Kanazawa Social Insurance Hospital, Kanazawa, Ishikawa, Japan; 6 Department of Gastroenterology, Hokuriku Hospital, Kanazawa, Ishikawa, Japan; 7 Department of Gastroenterology, Kanazawa Red Cross Hospital, Kanazawa, Ishikawa, Japan; 8 Department of Gastroenterology, Komatsu Municipal Hospital, Komatsu, Ishikawa, Japan; 9 Department of Internal Medicine, Suzu General Hospital, Suzu, Ishikawa, Japan; 10 Department of Internal Medicine, Sangane Clinic, Kouta, Aichi, Japan; 11 Department of Diagnostic Pathology, Ishikawa Prefectural Central Hospital, Kanazawa, Ishikawa, Japan; 12 Department of Gastroenterology, Kanazawa University Hospital, Kanazawa, Ishikawa, Japan; University Hospital Llandough, United Kingdom

## Abstract

**Objectives:**

Magnifying narrow-band imaging (M-NBI) is more accurate than white-light imaging for diagnosing small gastric cancers. However, it is uncertain whether moving M-NBI images have additional effects in the diagnosis of gastric cancers compared with still images.

**Design:**

A prospective multicenter cohort study.

**Methods:**

To identify the additional benefits of moving M-NBI images by comparing the diagnostic accuracy of still images only with that of both still and moving images. Still and moving M-NBI images of 40 gastric lesions were obtained by an expert endoscopist prior to this prospective multicenter cohort study. Thirty-four endoscopists from ten different Japanese institutions participated in the prospective multicenter cohort study. Each study participant was first tested using only still M-NBI images (still image test), then tested 1 month later using both still and moving M-NBI images (moving image test). The main outcome was a difference in the diagnostic accuracy of cancerous versus noncancerous lesions between the still image test and the moving image test.

**Results:**

Thirty-four endoscopists were analysed. There were no significant difference of cancerous versus noncancerous lesions between still and moving image tests in the diagnostic accuracy (59.9% versus 61.5%), sensitivity (53.4% versus 55.9%), and specificity (67.0% versus 67.6%). And there were no significant difference in the diagnostic accuracy between still and moving image tests of demarcation line (65.4% versus 65.5%), microvascular pattern (56.7% versus 56.9%), and microsurface pattern (48.1% versus 50.9%). Diagnostic accuracy showed no significant difference between the still and moving image tests in the subgroups of endoscopic findings of the lesions.

**Conclusions:**

The addition of moving M-NBI images to still M-NBI images does not improve the diagnostic accuracy for gastric lesions. It is reasonable to concentrate on taking sharp still M-NBI images during endoscopic observation and use them for diagnosis.

**Trial registration:**

Umin.ac.jp UMIN-CTR000008048

## Introduction

The magnifying narrow-band imaging (M-NBI) is useful in the differential diagnosis between early gastric carcinoma and gastritis [1]–[3], in determination of the lateral extent of differentiated gastric cancer to plan endoscopic therapy [4]–[6], and in visualization of clear contrast within the capillary pattern and the crypt pattern on the mucosa [7], [8]. M-NBI is more accurate for diagnosing gastric cancers than is conventional white-light imaging, magnifying white-light imaging, or magnifying chromoendoscopy [9], [10], [11]. Yao et al proposed a simple and systematic classification system (vessels plus surface classification system: VSCS) based on the microvascular pattern (MVP), microsurface pattern (MSP), and demarcation line (DL). If the endoscopic findings of M-NBI satisfy the diagnostic criteria of the VSCS, there is a very high possibility that the lesion is early carcinoma [12], [13], [14].

We usually observe gastric lesions with both moving and still images during endoscopic observation. M-NBI including moving images was shown to be more accurate than conventional white-light imaging for diagnosing small gastric mucosal cancers on site [15], [16]. Moving images can be thought of as a combination of a very large number of still images and provide an overwhelmingly greater amount of information compared with individual still images. In contrast, we usually use only still images for education, learning, and re-examination for diagnosis after observation because preparing moving images requires much more time and effort and because evaluating still images is simpler compared with the required playback of moving images. It is uncertain whether moving M-NBI images have any additional benefits in diagnosis compared with still images or whether learning to interpret moving M-NBI images is useful. We expected an additional effect of moving images in M-NBI diagnosis of cancer and non-cancer, and thus conducted this study. If there is a sufficient additional effect of moving images, it will be necessary to use moving images not only for diagnosis, but also for learning.

In this exploratory study, we compared the diagnostic accuracy of still M-NBI images only with that of both still and moving images, and attempted to identify the additional benefits of moving M-NBI images in terms of diagnostic accuracy.

## Patients/Materials and Methods

The protocols for this trial and supporting CONSORT checklist are available as supporting information; see Checklist S1 and Protocols S1 and S2.

### Study design and participating endoscopists

**Figure 1:**
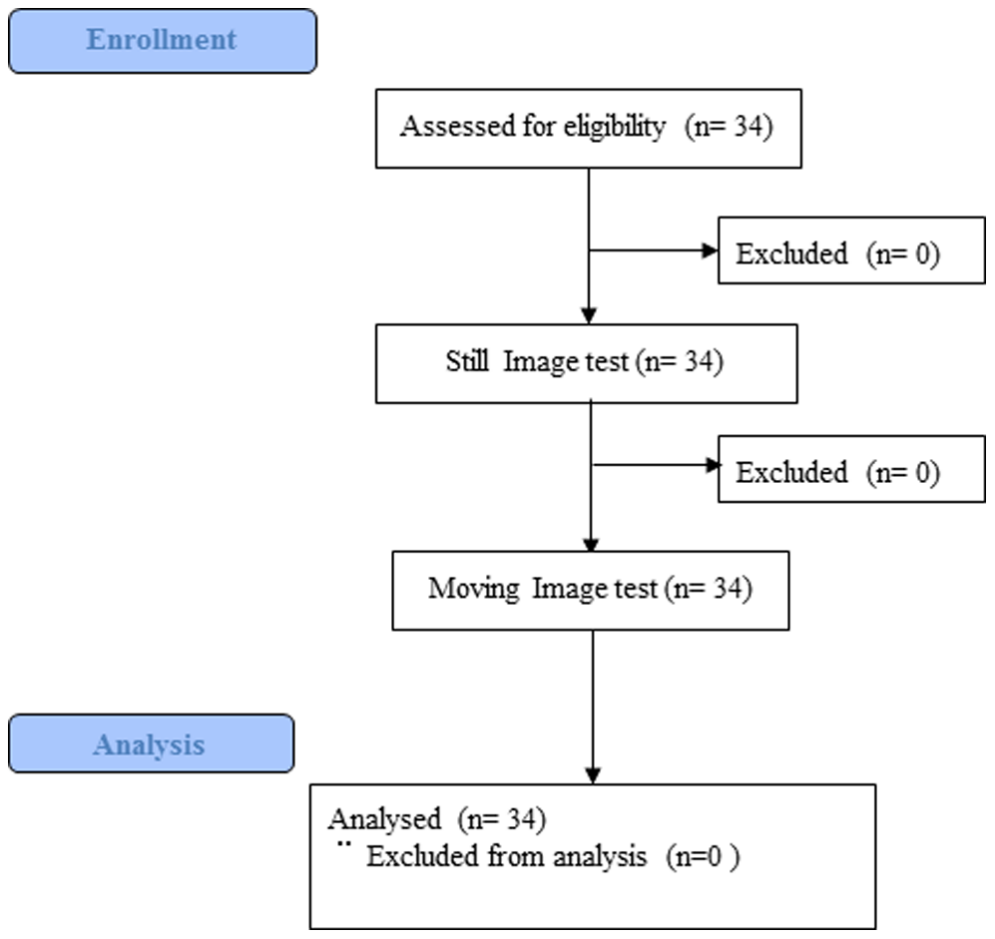
Flow chart of enrollment and analysis records.

This prospective multicenter study included the performances of 34 endoscopists with various levels of experience from 10 different institutions in Japan in accordance with the CONSORT 2010 Statement [17] and the Declaration of Helsinki. All participants aimed at mastery of the VSCS used in conjunction with M-NBI and were not limited in terms of their relative experience with the M-NBI procedure. Participants were first tested using still images only (still image test) within 2 weeks, and were then tested 1 month later using both still and moving images (moving image test) within 2 weeks (Figure 1). We evaluated the diagnostic accuracy, sensitivity, and specificity of both tests. Participants could observe the images any number of times within each answer period. They provided the following information by questionnaire before the study: board certification by the Japan Gastroenterological Endoscopy Society (JGES), years of experience with upper gastrointestinal endoscopy, number of upper gastrointestinal endoscopy procedures, number of M-NBI procedures, and number of M-NBI procedures in the previous month. We divided the participants into 2 groups and examined each using the questionnaire results in subgroup analysis.

### Endoscopy system

The subepithelial microvascular architecture and the mucosal microsurface structure can be visualized in high contrast in the NBI system [7], [8]. For magnification endoscopy with NBI and upper gastrointestinal tract magnifying endoscopy (GIF-H260Z; Olympus Medical Systems, Tokyo, Japan), a video processor (EVIS LUCERA Olympus CV-260SL; Olympus Medical Systems) and a light source (EVIS LUCERA Olympus CLV-260SL; Olympus Medical Systems) were used. The structure enhancement of the endoscopic video processor was set to B-mode level 8 for M-NBI. The color mode was fixed at level 1. To obtain stable endoscopic images at maximal magnification, a black soft hood (MAJ-1990 for GIF-H260Z; Olympus Medical Systems) was installed at the tip of the magnification endoscope. Moving images were recorded by a DVD recorder (Vardia RD-E304K; Toshiba, Tokyo, Japan).

### Endoscopic images

Still and moving M-NBI images of undiagnosed gastric lesions were recorded and evaluated based on the VSCS by an expert endoscopist (H.D.) prior to the study. We selected only M-NBI images without non-magnifying NBI and white-light endoscopy imaging to avoid affecting the endoscopists' judgment. Lesions with biopsy scars were not used for the test because the diagnosis may have been influenced by the scar. In all cases, the test results were considered to fulfill the VSCS diagnostic criteria. Also, the expert endoscopic diagnoses were consistent with the pathological diagnoses in all cases.

**Figure 2:**
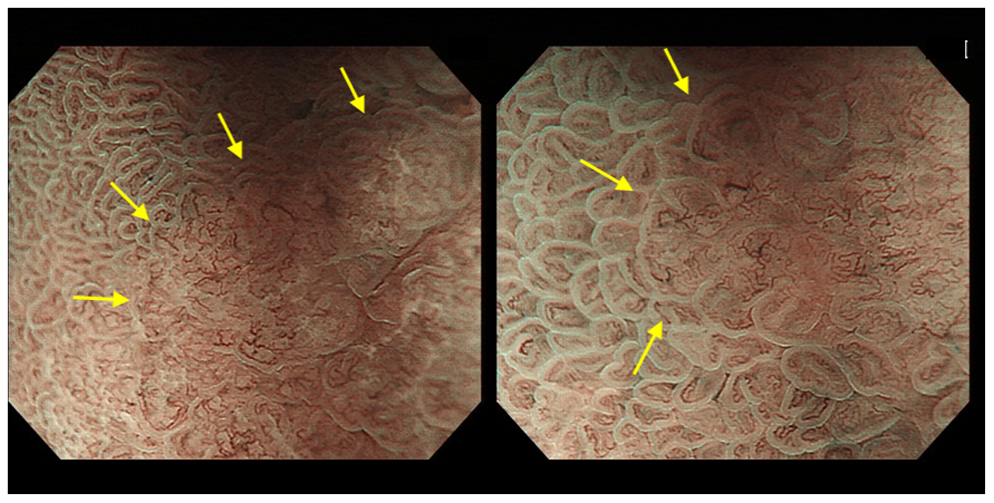
Cancerous lesion: Within a DL (arrows), irregular MVP and irregular MSP are noted.

**Figure 3:**
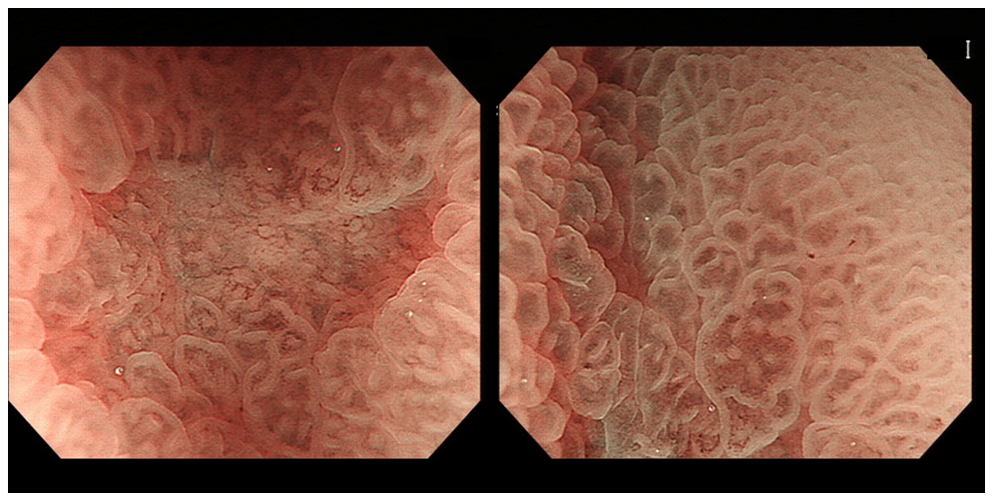
Non-cancerous lesion (gastritis): Absent DL, regular MVP, and regular MSP are noted.

The 40 questions of each test involved cancerous lesions (n = 21, 17 lesions were well differentiated adenocarcinoma dominant and 4 lesions were moderately differentiated adenocarcinoma dominant), noncancerous lesions (n = 19, 2 lesions were low-grade adenoma, 16 lesions were chronic gastritis, and 1 lesion was xanthoma), elevated lesions (n = 10), non-elevated (flat or depressed) lesions (n = 30), upper stomach lesions (n = 5), middle stomach lesions (n = 18), lower stomach lesions (n = 17), <10-mm lesions (n = 21), and ≥10-mm lesions (n = 19) (Table 1). The still image test included 5 to 8 M-NBI images, and the moving image test included 39 to 66 seconds per question. All pictures used for the still image test were contained in the moving image test and could be seen by pausing the moving image test. The moving images did not stop other than when viewed in the still image test. Figures 2 and 3 show representative endoscopic still images.

**Table 1:** Characteristics of 40 Gastric Lesions Used in Both Tests.

			No.	%
Histological type	Cancer		21	52.5
		Well differentiated adenocarcinoma	17	42.5
		Moderately differentiated adenocarcinoma	4	10.0
	Noncancer		19	47.5
		Low-grade adenoma	2	5.0
		Chronic gastritis	16	40.0
		Xanthoma	1	2.5
Elevation	Yes		10	25.0
	No		30	75.0
Tumor location	Upper		5	12.5
	Middle		18	45.0
	Lower		17	42.5
Size	<10 mm		21	52.5
	≥10 mm		19	47.5

### Diagnostic criteria for M-NBI

According to the diagnostic criteria of the VSCS, namely (1) the presence of an irregular MVP with a DL or (2) the presence of an irregular MSP with a DL, a case was defined as cancerous when at least one of the findings in (1) or (2) was present and as noncancerous when these findings were absent [12]. We set the endoscopic diagnostic grade according to certainty: grade 1, “noncancerous,” with a high degree of confidence; grade 2, “noncancerous,” with a low degree of confidence; grade 3, “indeterminate,” classified as “noncancerous”; grade 4, “cancerous,” with a low degree of confidence; grade 5, “cancerous,” with a high degree of confidence [14].

### Diagnosis of pathology

The diagnostic pathology criteria were based on the revised Vienna classification [18], [19]. Category 4 (C4) was defined by mucosal high-grade neoplasia; 4.1 by high-grade dysplasia/adenoma; 4.2 by noninvasive carcinoma (carcinoma in situ); and 4.3 by intramucosal carcinoma, and Category C5 was defined by submucosal tumor invasion, which were diagnosed as cancer. C1 (negative for neoplasia), C2 (indefinite for neoplasia), and C3 (mucosal low-grade neoplasia, or low-grade dysplasia/adenoma) were diagnosed as noncancerous lesions. Two pathologists assigned the postoperative pathological diagnoses, and the results were double-checked in all cases.

### Diagnosis of DL, MVP, and MSP

The diagnosis of DL, MVP, and MSP was determined by an expert endoscopist according to VSCS. DL was diagnosed as present or absent, and MVP and MSP were diagnosed as regular, irregular, or absent. DL of each test involved 30 present and 10 absent cases. MVP of each test involved 14 regular, 21 irregular, and 5 absent cases. MSP of each test involved 14 regular, 20 irregular, and 6 absent cases (Table 2).

**Table 2:** M-NBI findings of 40 Gastric Lesions Used in Both Tests.

		No.	%
Demarcation Line	Present	30	75.0
	Absent	10	25.0
Microvascular pattern	Regular	14	35.0
	Irregular	21	52.5
	Absent	5	12.5
Microsurface pattern	Regular	14	35.0
	Irregular	20	50.0
	Absent	6	15.0

### Outcome measures

The main outcome was a difference in the diagnostic accuracy of cancerous versus noncancerous lesions between the still image test and the moving image test. The secondary outcomes were (1) a difference in the sensitivity and specificity of the diagnosis of cancerous versus noncancerous lesions between both tests, (2) a difference in the accuracy of the diagnosis of DL, MVP, and MSP between both tests, (3) subgroup analysis of the endoscopic findings of the lesion, including macroscopic type, and (4) subgroup analysis of the characteristics of the endoscopists, including whether they were board-certified by the JGES and their relative experience with the M-NBI procedure.

### Statistical analysis

The paired t test was used to determine differences in diagnostic accuracy between the still image test and the moving image test. Pearson's chi-square test was used to determine differences in sensitivity and specificity between both tests. All statistical indices were determined to be significant at a *P* value of <0.05. The Benjamini–Hochberg procedure was used in subgroup analyses to reduce the chances of obtaining false-positive results. The endoscopic diagnostic grade according to the certainty of each test was evaluated using receiver operating characteristic (ROC) curve analysis, which correlates sensitivity and (1 – specificity). In addition, the difference in the area under the curve (AUC) values was determined by the paired t test. All analyses were performed with the use of SPSS statistical software version 10.5 J for Windows (SPSS Inc., Chicago, IL).

### Ethics statement

Written informed consent was obtained, and the institutional review board (IRB) of Ishikawa Prefectural Central Hospital approved the study. After that, IRB of each participating hospital (Saiseikai Kanazawa Hospital, Kanazawa Municipal Hospital, Kanazawa Medical Center, Kanazawa Social Insurance Hospital, KKR Hokuriku Hospital, Kanazawa Red Cross Hospital, Komatsu Municipal Hospital, Suzu General Hospital, Sangane Clinic, and Kanazawa University) approved the study. This study has been registered in the UMIN Clinical Trials Registry System as trial ID UMIN-CTR000008048.

## Results

**Figure 4:**
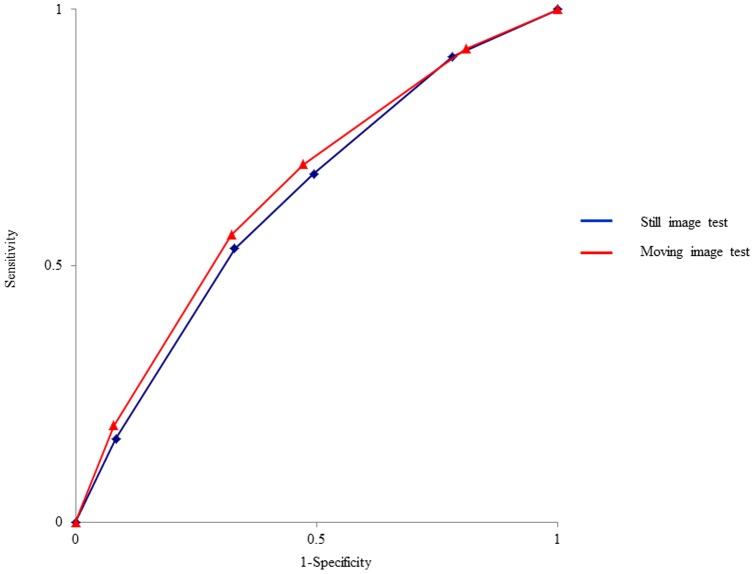
Receiver operating characteristic (ROC) curve of the endoscopic diagnostic grades is according to the certainty of each test correlated with sensitivity and (1 – specificity). The area under the curve (AUC) was as follows: still image test 0.629, moving image test 0.649 (*P* = 0.303).

Thirty-four endoscopists were analysed, and no endoscopists were excluded. There was no significant difference in the diagnostic accuracy of cancerous versus noncancerous lesions between the still and moving image tests (59.9% versus 61.5%, respectively; *P* = 0.272). There were also no significant differences in the sensitivity (53.4% versus 55.9%, respectively; *P* = 0.312) or specificity (67.0% versus 67.6%, respectively; *P* = 0.812) of the diagnosis of cancerous versus noncancerous lesions between the still and moving image tests (Table 3, Table S1). The raw data in each endoscopist and each question were shown in Table S2 (still image test) and Table S3 (moving image test). The raw data of endoscopic experience and diagnostic accuracy in each endoscopist were shown in Table S4. And the raw data of diagnostic accuracy in each question were shown in Table S5. Table 4 shows the endoscopic diagnoses for all lesions according to the degree of certainty in the still and moving image tests. ROC curves were generated as shown in Figure 4. No significant difference was found between the AUC values of the still and moving image tests (0.629 versus 0.649, respectively; *P* = 0.303).

**Table 3:** Diagnostic Accuracy, Sensitivity, and Specificity of Cancerous versus Noncancerous Lesions of Still Image Test versus Moving Image Test in Overall 34 Endoscopists.

	Still Image Test, % [95% C.I.]	Moving Image Test, % [95% C.I.]	*P* value
Accuracy	59.9 [56.1–63.6]	61.5 [57.1–65.8]	.272
Sensitivity	53.4 [48.0–58.7]	55.9 [49.2–62.6]	.312
Specificity	67.0 [61.9–72.1]	67.6 [61.4–73.8]	.812

**Table 4:** Endoscopic Diagnoses for All Lesions according to Grade of Certainty in Still and Moving Image Tests.

	Still Image Test			Moving Image Test		
Grade*	Cancer	Non-cancer	Total	Cancer	Non-cancer	Total
1	66	141	207	55	123	178
2	163	186	349	161	218	379
3	104	106	210	98	96	194
4	265	159	424	265	158	423
5	116	54	170	135	51	186
Total	714	646	1360	714	646	1360

*Grade 1: “noncancerous,” with high degree of confidence; grade 2: “noncancerous,” with low degree of confidence; grade 3: “indeterminate,” classified as “noncancerous”; grade 4: “cancerous,” with low degree of confidence; grade 5: “cancerous,” with high degree of confidence.

There were no significant differences in the diagnostic accuracy of DL (65.4% versus 65.5%, respectively; *P* = 0.955), MVP (56.7% versus 56.9%, respectively; *P* = 0.866), or MSP (48.1% versus 50.9%, respectively; *P* = 0.115) (Table 5, Table S6).

**Table 5:** Diagnostic Accuracy of Vessels Plus Surface Classification System in Still Image Test versus Moving Image Test by All 34 Endoscopists.

	Accuracy, % [95% C.I.]		*P* value
	Still Image Test	Moving Image Test	
Demarcation Line	65.4 [62.0–68.9]	65.5 [61.4–69.6]	.955
Microvascular pattern	56.7 [52.7–60.8]	56.9 [53.2–60.6]	.866
Microsurface pattern	48.1 [44.5–51.8]	50.9 [46.6–55.1]	.115

Diagnostic accuracy showed no significant difference between the still and moving image tests in the subgroups of endoscopic findings of the lesions. There was no significant difference in the macroscopic type of elevated lesions (57.9% versus 60.3%, respectively; *P* = 0.389), nonelevated lesions (60.5% versus 61.9%, respectively; *P* = 0.405), upper stomach lesions (65.3% versus 67.1%, respectively; *P* = 0.646), middle stomach lesions (57.7% versus 59.2%, respectively; *P* = 0.630), lower stomach lesions (60.6% versus 62.3%, respectively; *P* = 0.538), <10-mm lesions (62.6% versus 65.8%, respectively; *P* = 0.228), and ≥10-mm lesions (56.8% versus 56.7%, respectively; *P* = 0.951) (Table 6, Table S7).

**Table 6:** Diagnostic Accuracy of Still Image Test versus Moving Image Test in the Subgroups of Endoscopic Findings.

		Accuracy, % [95% C.I.]		*P* value
Subgroup		Still Image Test	Moving Image Test	
Elevation	Yes (n = 10)	57.9 [40.6–75.3]	60.3 [46.6–74.0]	.389
	No (n = 30)	60.5 [52.6–68.4]	61.9 [53.8–69.9]	.405
Tumor location	Upper (n = 5)	65.3 [38.8–91.8]	67.1 [45.5–88.6]	.646
	Middle (n = 18)	57.7 [47.6–67.7]	59.2 [48.6–69.7]	.630
	Lower (n = 17)	60.6 [48.1–73.0]	62.3 [51.1–73.5]	.538
Size	<10 mm (n = 21)	62.6 [53.6–71.6]	65.8 [57.3–74.4]	.228
	≥10 mm (n = 19)	56.8 [45.3–68.3]	56.7 [46.0–67.3]	.951

Diagnostic accuracy was lower in the still image test than that in the moving image test among the subgroup of endoscopists who were board-certified by the JGES (62.7% versus 66.5%, respectively; *P* = 0.041), the subgroup of endoscopists who had experienced more than 200 M-NBI procedures (65.6% versus 68.9%, respectively; *P* = 0.049), and the subgroup of endoscopists who had experienced more than 15 M-NBI procedures in the previous month (65.1% versus 69.3%, respectively; *P* = 0.037). However, after applying the Benjamini–Hochberg procedure, the significant differences disappeared. Diagnostic accuracy showed no significant differences between the subgroups (the number of upper gastrointestinal endoscopy procedures and the years of experience of upper gastrointestinal endoscopy) (Table 7, Table S8).

**Table 7:** Diagnostic Accuracy of Still Image Test versus Moving Image Test in the Subgroups of Endoscopist Characteristics.

		Accuracy, % [95% C.I.]		*P* value
Subgroup		Still Image Test	Moving Image Test	
Board-Certified by JGES*	Yes (n = 15)	62.7 [58.5–66.8]	66.5 [62.8–70.2]	.041
	No (n = 19)	57.8 [51.9–63.6]	57.5 [50.5–64.5]	.908
Years of experience performing upper gastrointestinal endoscopy	≥8 (n = 17)	62.6 [59.2–66.1]	65.2 [61.2–69.4]	.161
	<8 (n = 17)	57.2 [50.5–63.9]	57.6 [49.9–65.4]	.856
Number of cases of upper gastrointestinal endoscopy	≥5000 (n = 20)	61.9 [57.1–66.6]	64.5 [59.8–69.2]	.112
	<5000 (n = 14)	57.1 [50.9–63.4]	57.1 [48.7–65.6]	>.999
Number of cases of M-NBI procedures	≥200 (n = 18)	65.6 [60.4–70.7]	68.9 [64.2–73.6]	.049
	<200 (n = 16)	53.6 [50.1–57.1]	53.1 [47.7–58.5]	.858
Number of cases of M-NBI procedures in the previous month	≥15 (n = 15)	65.2 [58.8–71.5]	69.3 [63.2–75.5]	.037
	<15 (n = 19)	55.7 [52.0–59.5]	55.3 [50.5–60.0]	.811

*Japan Gastroenterological Endoscopy Society.

## Discussion

In practice, we observe a gastric lesion with moving M-NBI images and reappraise the lesion with still images after the observation. That is to say, we usually diagnose the lesion by utilizing both still and moving images. A moving image is an aggregate of a very large number of still images. The still images provide less information compared with a moving image. As an M-NBI learning tool, preparing moving images requires much more time and effort compared with preparing only still images [20], [21], and checking still images is simple compared with the playback required of moving images [20]; thus, we usually use only still images. In general, there are almost no records of moving images when an observer wants to recheck the lesion and when endoscopists other than the observer want to diagnose the lesion. In contrast, with the widespread availability of Internet resources, learning programs with web-based videos are becoming a great tool to improve the diagnostic yield with endoscopy [22], [23], [24]. To our knowledge, the difference between still images and moving images has not been adequately evaluated. For ulcerative colitis, for example, the mucosal inflammation might be documented nearly as well with a still image as on a video clip, and so systematic use of still images probably improves the endoscopy reports by adding more objective information about the mucosal inflammation [20]. Therefore, we performed the present study to clarify whether only still images are adequate or whether the addition of moving images is required in M-NBI.

In this multicenter study, there were no significant differences in the diagnostic accuracy, sensitivity, and specificity of cancerous versus noncancerous lesions between the still and moving image tests, and the diagnostic superiority of moving images over still images could not be proved. In addition, ROC curves of the two tests were almost identical. Diagnostic accuracy did differ in some subgroup analyses, but the differences were very small and disappeared after applying the Benjamini–Hochberg procedure. Even if the diagnostic superiority of moving images is proven in a future confirmatory trial with a greater number of endoscopists experienced in M-NBI, there may be not enough clinical significance because of the small differences between the 2 tests.

Based on the results of this study, it is likely that sharp still images with the evident feature of the lesion have sufficient information for equivalence with moving images. It is reasonable to concentrate on taking high-quality still images at the time of observation by M-NBI and to obtain a diagnosis from the still images at the time of or after the observation.

It is necessary, however, to consider the use of M-NBI images as a study tool for VSCS learning. It will be important to adequately understand each endoscopic finding of DL, MVP, and MSP when learning VSCS [25], [26]. In this multicenter study, there were also no significant differences in the accuracy of the diagnosis of DL, MVP, and MSP between the 2 tests. As a learning tool, there may not be any advantages of moving images. Still images may facilitate better recognition and understanding because they do not move. Moreover, given the large amount of time, effort, and cost associated with preparation of moving images, still images may be preferable to moving images [20]. When individuals learn to interpret M-NBI, still images alone are expected to be an adequate learning tool [26], [27], [28].

In this study, the high-quality images were obtained by an expert endoscopist, not by the study participants, and we did not evaluate the recording technique used by the participants. In M-NBI, specific techniques are required to accurately evaluate and record images [26], [27]. Based on these points, we should consider that the participants' diagnostic accuracy in practice may not agree with the results of this study. Diagnosis using M-NBI cannot be performed well without a photographing technique [26]. We should also aim to further improve photographing techniques.

This study has some limitations. First, no formal sample size was estimated because this study was conducted as an exploratory study. Second, it was a single-arm trial involving a moving image test after a still image test. Third, only one endoscopist evaluated the M-NBI results; thus, the answers to each item (DL, MVP, and MSP) of the VSCS may not be completely accurate. Finally, learning and experience of M-NBI may have been acquired in a 1-month interval of both tests.

In conclusion, our results indicate that the addition of moving M-NBI images to still M-NBI images does not improve the diagnostic accuracy of gastric lesions. Thus focusing on taking sharp still M-NBI images during endoscopic observation and using them for diagnosis is reasonable.

## Supporting Information

Table S1 **Correspond to Table 3.**
(XLSX)

Table S2 **Raw data in each endoscopist and each question (still image test).**
(XLSX)

Table S3 **Raw data in each endoscopist and each question (moving image test).**
(XLSX)

Table S4 **Raw data of endoscopic experience and diagnostic accuracy in each endoscopist.**
(XLSX)

Table S5 **Raw data of diagnostic accuracy in each question.**
(XLSX)

Table S6 **Correspond to Table 5.**
(XLSX)

Table S7 **Correspond to Table 6.**
(XLSX)

Table S8 **Correspond to Table 7.**
(XLSX)

Checklist S1 **CONSORT checklist.**
(DOC)

Protocol S1 **Trial protocol (English).**
(DOCX)

Protocol S2 **Trial protocol (Japanese).**
(DOCX)
