# Supplementary material for: Assessment of Still and Moving Images in the Diagnosis of Gastric Lesions Using Magnifying Narrow-Band Imaging in a Prospective Multicenter Trial
Source: PLoS One. 2014 Jul 2;9(7):e100857. doi: 10.1371/journal.pone.0100857 (PMC4079511; doi:10.1371/journal.pone.0100857)
Supplement: Protocol S1 — Trial protocol (English). (DOCX) [file pone.0100857.s010.docx]

Assessment of Still and Moving Images in the Diagnosis of Gastric Lesions Using Magnifying Narrow-Ban​d Imaging in a Prospectiv​e Multicente​r Trial
**（NBI-SM Study）**

**(UMIN000008048)**

Protocol

Doyama Hisashi,
Tomoyuki Hayashi

Department of Gastroenterology, Ishikawa Prefectural Central Hospital, Ishikawa, Japan

E-mail：[hayasix0917@gmail.com](mailto:hayasix0917@gmail.com)

1. Objectives

2. Study Design

3. Patients/Materials and Methods

4. Diagnostic criteria for M-NBI

5. Statistical analysis

6. Ethics statement

7. Report of the Results

8. Research Institution

9. References

1. Objectives

The magnifying narrow-band imaging (M-NBI) is useful in the differential diagnosis between early gastric carcinoma and gastritis,^【1-3】^ in determination of the lateral extent of differentiated gastric cancer to plan endoscopic therapy,^【4-6】^ and in visualization of clear contrast within the capillary pattern and the crypt pattern on the mucosa.^【7,8】^ Yao et al proposed a simple and systematic classification system (vessels plus surface classification system: VSCS) based on the microvascular pattern (MVP), microsurface pattern (MSP), and demarcation line (DL). If the endoscopic findings of M-NBI satisfy the diagnostic criteria of the VSCS, there is a very high possibility that the lesion is early carcinoma.^【9,10】^

We usually observe gastric lesions with both moving and still images during endoscopic observation. M-NBI including moving images was shown to be more accurate than conventional white-light imaging for diagnosing small gastric mucosal cancers on site.^【11】^ Moving images can be thought of as a combination of a very large number of still images and provide an overwhelmingly greater amount of information compared with individual still images. In contrast, we usually use only still images for education, learning, and re-examination for diagnosis after observation because preparing moving images requires much more time and effort and because evaluating still images is simple compared with the required playback of moving images. It is uncertain whether moving M-NBI images have any additional benefits in diagnosis compared with still images or whether learning to interpret moving M-NBI images is useful. We expected an additional effect of moving images in M-NBI diagnosis of cancer and non-cancer, and thus conducted this study. If there is a sufficient additional effect of moving images, it will be necessary to use moving images not only for diagnosis, but also for learning.

In this exploratory study, we compared the diagnostic accuracy of still M-NBI images only with that of both still and moving images, and attempted to identify the additional benefits of moving M-NBI images in terms of diagnostic accuracy.

2. Study Design


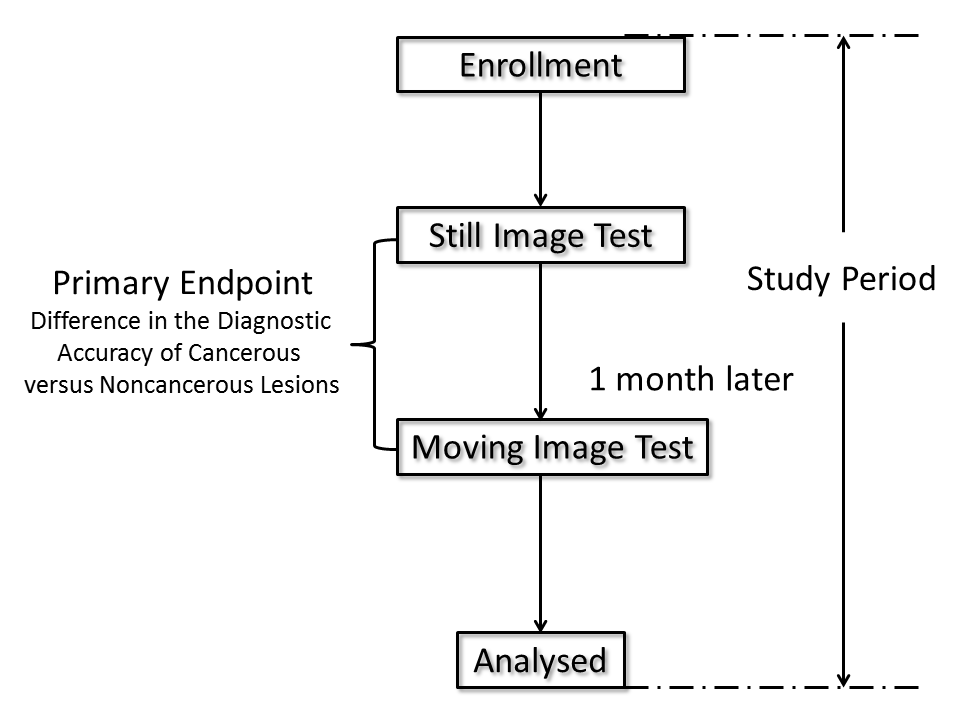


Participants were first tested using still images only (still image test) within 2 weeks, and were then tested 1 month later using both still and moving images (moving image test) within 2 weeks. We evaluated the diagnostic accuracy, sensitivity, and specificity of both tests. Participants could observe the images any number of times within each answer period. They provided the following information by questionnaire before the study: board certification by the Japan Gastroenterological Endoscopy Society (JGES), years of experience with upper gastrointestinal endoscopy, number of upper gastrointestinal endoscopy procedures, number of M-NBI procedures, and number of M-NBI procedures in the previous month. We divided the participants into 2 groups and examined each using the questionnaire results in subgroup analysis.

The main outcome was a difference in the diagnostic accuracy of cancerous versus noncancerous lesions between the still image test and the moving image test. The secondary outcomes were (1) a difference in the sensitivity and specificity of the diagnosis of cancerous versus noncancerous lesions between both tests, (2) a difference in the accuracy of the diagnosis of DL, MVP, and MSP between both tests, (3) subgroup analysis of the endoscopic findings of the lesion, including macroscopic type, and (4) subgroup analysis of the characteristics of the endoscopists, including whether they were board-certified by the JGES and their relative experience with the M-NBI procedure.

3. Patients/Materials and Methods

The subepithelial microvascular architecture and the mucosal microsurface structure can be visualized in high contrast in the NBI system.^【7,8】^ For magnification endoscopy with NBI and upper gastrointestinal tract magnifying endoscopy (GIF-H260Z; Olympus Medical Systems, Tokyo, Japan), a video processor (EVIS LUCERA Olympus CV-260SL; Olympus Medical Systems) and a light source (EVIS LUCERA Olympus CLV-260SL; Olympus Medical Systems) were used. The structure enhancement of the endoscopic video processor was set to B-mode level 8 for M-NBI. The color mode was fixed at level 1. To obtain stable endoscopic images at maximal magnification, a black soft hood (MAJ-1990 for GIF-H260Z; Olympus Medical Systems) was installed at the tip of the magnification endoscope. Moving images were recorded by a DVD recorder (Vardia RD-E304K; Toshiba, Tokyo, Japan).

Still and moving M-NBI images of undiagnosed gastric lesions were recorded and evaluated based on the VSCS by an expert endoscopist prior to the study. We selected only M-NBI images without non-magnifying NBI and white-light endoscopy imaging to avoid affecting the endoscopists’ judgment. Lesions with biopsy scars were not used for the test because the diagnosis may have been influenced by the scar. In all cases, the test results were considered to fulfill the VSCS diagnostic criteria. Also, the expert endoscopic diagnoses were consistent with the pathological diagnoses in all cases.

All pictures used for the still image test were contained in the moving image test and could be seen by pausing the moving image test. The moving images did not stop other than when viewed in the still image test.

As a general rule, all physicians who wish to participate will be allowed to participate.

4. Diagnostic criteria for M-NBI

According to the diagnostic criteria of the VSCS, namely (1) the presence of an irregular MVP with a DL or (2) the presence of an irregular MSP with a DL, a case was defined as cancerous when at least one of the findings in (1) or (2) was present and as noncancerous when these findings were absent.^【9】^

5. Statistical analysis

The paired t test was used to determine differences in diagnostic accuracy between the still image test and the moving image test. Pearson’s chi-square test was used to determine differences in sensitivity and specificity between both tests. All statistical indices were determined to be significant at a *P* value of <0.05. Bonferroni correction was used in subgroup analyses to reduce the chances of obtaining false-positive results. All analyses were performed with the use of SPSS statistical software version 10.5J for Windows (SPSS Inc., Chicago, IL).

6. Ethics statement

Written informed consent was obtained, and the institutional review board (IRB) of each institution approved the study. This study has been registered in the UMIN Clinical Trials Registry System as trial ID UMIN-CTR000008048.

7. Report of the Results

Research leader can decide the author of the paper in consultation with representatives of each institution. The main results are posted to the paper in English after the final analysis.

8. Research Institution

^1^ Ishikawa Prefectural Central Hospital, Ishikawa, Japan

^2^ Saiseikai Kanazawa Hospital, Ishikawa, Japan

^3^ Kanazawa Municipal Hospital, Ishikawa, Japan

^4^ Kanazawa Medical Center, Ishikawa, Japan

^5^ Kanazawa Social Insurance Hospital, Ishikawa, Japan

^6^ KKR Hokuriku Hospital, Ishikawa, Japan

^7^ Kanazawa Red Cross Hospital, Ishikawa, Japan

^8^ Komatsu Municipal Hospital, Ishikawa, Japan

^9^ Suzu General Hospital, Ishikawa, Japan

^10^ Sangane Clinic, Aichi, Japan

9. References

1. Yao K, Oishi T, Matsui T, et al. (2002) Novel magnified endoscopic findings of microvascular architecture in intramucosal gastric cancer. Gastrointest Endosc 56: 279-284.
2. Yao K, Iwashita A, Tanabe H, et al. (2007) Novel zoom endoscopy technique for diagnosis of small flat gastric cancer: a prospective, blind study. Clin Gastroenterol Hepatol 5: 869-878.
3. Kato M, Kaise M, Yonezawa J, et al. (2010) Magnifying endoscopy with narrow-band imaging achieves superior accuracy in the differential diagnosis of superficial gastric lesions identified with white-light endoscopy: a prospective study. Gastrointest Endosc 72: 523-529.
4. Yao K, Yao T, Iwashita A. (2002) Determining the horizontal extent of early gastric carcinoma: two modern techniques based on differences in the mucosal microvascular architecture and density between carcinoma and non-carcinomatous mucosa. Dig Endosc 14: S83-87.
5. Nagahama T, Yao K, Maki S, et al. (2011) Usefull of magnifying endoscopy with narrow-band imaging for determining the horizontal extent of early gastric cancer when there is an unclear margin by chromoendoscopy. Gastrointest Endosc 75: 1259-1267.
6. Kiyotoki S, Nishikawa J, Satake M, et al. (2010) Usefulness of magnifying endoscopy with narrow-band imaging for determining gastric tumor margin. J Gastroenterol Hepatol 25: 1636-1641.
7. Gono K, Yamazaki K, Doguchi N, et al. (2003) Endoscopic observation of tissue by narrow band illumination. Opt Rev 10: 211-215.
8. Gono K, Obi T, Yamaguchi M, et al. (2004) Appearance of enhanced tissue features in narrow-band endoscopic imaging. J Biomed Opt. 9: 568-577.
9. Yao K, Anagnostopoulos GK, Ragunath K. (2009) Magnifying endoscopy for diagnosing and delineating early gastric cancer. Endoscopy 41: 462-467.
10. Miwa K, Doyama H, Ito R, et al. (2012) Can magnifying endoscopy with narrow band imaging be useful for low grade adenomas in preoperative biopsy specimens. Gastric Cancer 15: 170–178.
11. Ezoe Y, Muto M, Uedo N, et al. (2011) Magnifying narrowband imaging is more accurate than conventional white-light imaging in diagnosis of gastric mucosal cancer. Gastroenterology 141: 2017-2025.
