# Supplementary material for: Assessment of Still and Moving Images in the Diagnosis of Gastric Lesions Using Magnifying Narrow-Band Imaging in a Prospective Multicenter Trial
Source: PLoS One. 2014 Jul 2;9(7):e100857. doi: 10.1371/journal.pone.0100857 (PMC4079511; doi:10.1371/journal.pone.0100857)
Supplement: Protocol S2 — Trial protocol (Japanese). (DOCX) [file pone.0100857.s011.docx]

**『Narrow-band imaging併用拡大内視鏡(M-NBI)観察を用いた胃病変の
質的診断における静止画(Still image)と動画(Moving image)の診断能の検討』に関する臨床試験実施計画書**

**（略称：NBI-SM Study）**

**The difference of diagnostic ability between still image and moving image in qualitative diagnosis of the gastric lesion using magnifying narrow-band imaging endoscopy**

(UMIN000008048)

作成日：　2012年　7月　1日

研究代表者

石川県立中央病院　消化器内科　土山　寿志

〒920-8530　石川県金沢市鞍月東2丁目1番地

TEL：076-237-8211

FAX：076-238-2337

E-mail：doyama.134@ipch.jp

研究事務局

石川県立中央病院　消化器内科　林　智之

E-mail：hayasix0917@gmail.com

**目次**

1. **研究の背景**
2. **研究の目的**
3. **研究の方法**

(1)　研究のデザイン

(2)　研究のアウトライン

(3)　参加医師の条件

(4)　事前アンケート

(5)　質的診断テスト

(6)　研究期間

1. **M-NBI所見の判定**
2. **評価項目**
3. **倫理的事項**
4. **研究組織**
5. **結果の報告**
6. **参考文献**

**1. 研究の背景**

小さくまたは平坦な早期胃癌は，癌に特徴的な肉眼像を呈しないために、従来の内視鏡では，診断が困難であった．八尾らは、早期胃癌の新しい拡大内視鏡診断法を開発し、早期胃癌に特徴的な微小血管構築像を報告した[1]。同時に、（１）従来より不可能であった限局した胃炎と表面平坦型胃癌や微小癌との鑑別診断[2-4]、（２）内視鏡治療に必要な境界不明瞭な分化型癌の境界診断[5,6]に有用であると報告した。

これに加え、narrow-band imaging (NBI)という画像強調処理を用いた技術が内視鏡観察に応用され、本機能を有する電子内視鏡システムがすでに実際の内視鏡検査に使用されている。管腔臓器の中でも胃は、管腔が広いためにNBIを非拡大観察に併用すると画像が暗くなり実用に耐えないため、胃粘膜の観察には近接拡大観察にのみにNBIは併用される（NBI併用拡大内視鏡：magnifying narrow-band imaging, M-NBI））。NBIを胃拡大内視鏡に併用すると、白色光拡大観察と比較し、粘膜表層の微小血管構築像のコントラストが高くなり、粘膜表面微細構造も視覚化できるなどの有用性が報告されている[7]。さらに八尾らは、上皮下の毛細血管・集合細静脈・病的な微小血管を微小血管構築像(microvascular pattern, MVP)として、腺窩辺縁上皮・粘膜白色不透明物資を粘膜表面微細構造(microsurface pattern, MSP)として視覚化し、MVPおよびMSPの規則性と病変部と非病変部との境界(demarcation line, DL)の有無を指標としたVS classification systemが、癌と非癌の鑑別に有用であることを報告した[8,9]。

最近多施設共同研究により、我々は白色光内視鏡観察(conventional white-light imaging, C-WLI)と、M-NBIのランダム化比較試験にて、動的観察を含むM-NBIが胃小陥凹病変に対する高い診断能を有することを報告した[10]。また、静止画による胃病変診断テストを用いることによりM-NBI診断能が向上する可能性及び、静止画による教育の有用性を報告した[11]。しかし、静止画における判定ではDemarcation Line(DL)の正診率が43.6～48.9%と低く、静止画でのDL診断の限界が示唆された[11]。この問題に対処するため、DLの有無を判断しやすいと考えられる動画を用いたテストによる診断能の更なる向上の検討が期待される。また実臨床では病変を動画にて観察し、検査終了後に静止画にて病変の再評価を行っており、静止画単独の評価のみでは実臨床に即さない可能性も考えられる。

動画を使用した胃病変診断テストの検討は極めて乏しく、今後のM-NBI教育の方向性を探求するため、本研究を企画した。

**２．研究の目的**

胃病変に対するM-NBIの質的診断能における、静止画に動画を付加することによる診断能の上乗せ効果を明らかにする。

**３. 研究の方法**

(1)　研究のデザイン

登録した内視鏡医は、まずM-NBIに関する静止画の質的診断テスト(静止画テスト)を受験し、その解答締切の2週間後に、M-NBIに関する動画の質的診断テスト(動画テスト)を受験する。

(2)　研究のアウトライン

　　　　　　　　　
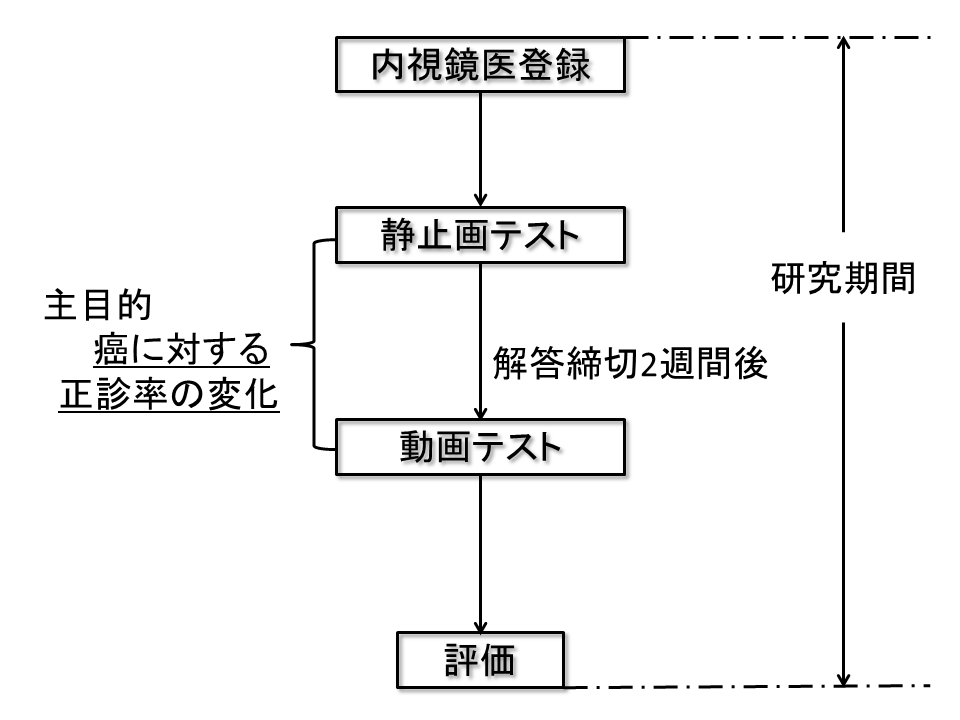


（１）主要評価項目：静止画テスト、動画テストでの正診率の変化

（２）副次的評価項目

1)静止画テスト、動画テストでの感度、特異度の変化

2)静止画テスト、動画テストにおけるMVP、MSP、DLの正診率、感度、特異度の変化

3)主要評価項目および副次的評価項目1)2)において、

肉眼型・色調別、専門医・非専門医別、上部消化管内視鏡検査の経験数別、
M-NBIの経験数別、M-NBIの月間経験数別　を層別因子とした、
静止画・動画の癌に対する正診率・感度・特異度の変化

(3)　参加医師の条件

本研究は、熟練者から初学者まで幅広い参加を望むため、内視鏡経験数やM-NBI経験数などでの参加医師の限定はしないが、VS classification systemを診断基準とするNBI併用拡大観察を実践しているものとする。

(4)　事前アンケート

参加医師は事前に以下のことを申告することとする。

１．日本内視鏡学会専門医の有無

２．上部消化管内視鏡経験年数

３．上部消化管内視鏡経験数

４．M-NBIの経験数

５．アンケート記載時の直前1ヶ月間の月間M-NBIの経験数

６．Full ZoomによるNBI拡大観察施行の有無

(5)　質的診断テスト

　2009年11月から2012年4月まで、石川県立中央病院にて研究代表者が施行した内視鏡検査にて撮影した病変の中で、病理組織が判明している40病変を静止画・動画をテストに用いる。

M-NBI画像は、NBI併用電子内視鏡システムとして、オリンパスメディカルシステムズ(株)社製EVIS LUCERA ビデオシステムセンターCV-260SL、高輝度光源装置CLV-260SLと光学拡大機能付き上部消化管ビデオスコープGIF-H260Zを用いる。ビデオシステムセンターの構造強調機能設定は、B mode レベル8、色彩強調1に設定する。拡大ビデオスコープの先端には拡大観察用のblack soft hood（MAJ-1990）を装着して使用する。

静止画テストに用いるM-NBI画像は、最大倍率で撮影された静止画の中で、その病変におけるDL、MVP、MSPの特徴をとらえているものを、1病変につき5-8枚選択する。

動画テストには、病変の全体像が入らず、静止画テストで選択された画像が全て含まれることを必要条件として編集した動画を使用する。さらに動画の病変に対応した静止画テストに用いた静止画も閲覧可能とする。

持ち越し効果のバイアスを避けるためにC-WLIによる画像は呈示しない。

診断の精度に影響する可能性があるため、可能な限り生検痕を含む画像は使用しない。

癌/非癌の正診は2名以上の病理専門医の病理組織学的所見とし、DL、MVP、MSPの正診は研究代表者、研究事務局の所見とする。

(6)　研究期間

登録締め切りから、静止画テストの解答期間を2週間とし、その2週間後に動画テストの解答期間を2週間設ける。したがって研究期間は6週間となる。何らかの障害で研究期間の延長を余儀なくされた場合は、その理由を十分に調査し、その対策を考慮した上で、必要があれば再度倫理委員会に申請して研究期間を延長する。

**４．M-NBI所見の判定　～M-NBI診断基準～**

八尾らのVS classification system[8,9]に基づき以下の3項目を判定する。

１．demarcation line (DL)：病変部と非病変部との明瞭な境界

　　　　　判定　present / absent / indeterminate

２．microvascular pattern (V)：微小血管構築像

　　　　　判定　regular / irregular / absent / indeterminate

３．microsurface pattern (S)：表面微細構造

　　　　　判定　regular / irregular / absent / indeterminate

DL-presentかつV-irregular、もしくはDL-presentかつS-irregularがあれば癌、それ以外を非癌と判定する。本試験ではその確信度別に以下のgrade 1～5に分類する。

grade 1　M-NBIにて非癌と診断できる病変。

　grade 2　M-NBIにて非癌を疑うが、確定診断のために生検が必要な病変。

　grade 3　M-NBIでは癌・非癌の判定が困難で、確定診断のために生検が必要な病変。

　　※ただし、M-NBIにて腺腫(LGA)と診断できるものはgrade 3-LGAとして記載する。

　grade 4　M-NBIにて癌を疑うが、確定診断のために生検が必要な病変。

　grade 5　M-NBIにて癌と診断できる病変。

最終的な内視鏡診断は、grade 1～3を非癌、grade 4～5を癌とする。

　M-NBI所見とgrade分類の組合せを表1に示す。M-NBI所見とgrade分類に乖離がみられる場合はM-NBI所見を重視し、grade分類を変更して登録する。

表1　 VS classification systemとgrade分類との対応

| DL | V | S | grade分類 |
| --- | --- | --- | --- |
| present | regular | regular | 1、2、3-LGA |
|  |  | irregular | 4、5 |
|  |  | absent | 1、2、3-LGA |
|  |  | indeterminate | 1、2、3 |
|  | irregular | regular  irregular  absent  indeterminate | 4、5 |
|  | absent | regular | 1、2、3-LGA |
|  |  | irregular | 4、5 |
|  |  | absent/indeterminate | 3 |
|  | indeterminate | regular | 3 |
|  |  | irregular | 4、5 |
|  |  | absent/indeterminate | 3 |
| absent | regular  irregular  absent  indeterminate | regular  irregular  absent  indeterminate | 1、2 |
| indeterminate | regular  irregular  absent  indeterminate | regular  irregular  absent  indeterminate | 3 |

**５．評価項目**

（１）主要評価項目：静止画テスト、動画テストでの正診率の変化

設定の根拠：質的診断能の指標として最も重要なものは癌・非癌の診断であり、
感度・特異度の両方を評価できる正診率を設定する。
静止画テスト・動画テストの正診率の差は、対応のないt検定を用いて
比較する。

（２）副次的評価項目

1)静止画テスト、動画テストでの感度、特異度の変化

2)静止画テスト、動画テストにおけるMVP、MSP、DLの正診率、感度、特異度の変化

3)主要評価項目および副次的評価項目1)2)において、

肉眼型・色調別、専門医・非専門医別、上部消化管内視鏡検査の経験数別、
M-NBIの経験数別、M-NBIの月間経験数別　を層別因子とした、
静止画・動画の癌に対する正診率・感度・特異度の変化

**６．倫理的事項**

(1）患者の保護

すべての研究はヘルシンキ宣言、臨床研究に関する倫理指針(厚生労働省)に従って実施し、被験者の人権保護に努める。

(2）プライバシーの保護

本試験は、患者の氏名（イニシャルを含む）、年齢などの個人情報は、研究の対象となる画像や研究結果には、一貫してまったく表示させない。検者は全員守秘義務が法的に課せられている医師であり、内視鏡画像を検者以外には見せないことについて文書で確約を得る。

(3）プロトコールの遵守

本研究に参加する研究者は、患者の安全と人権を損なわない限りにおいて本研究実施計画書を遵守する。

(4）倫理審査委員会の承認

本試験は、石川県立中央病院の倫理委員会の承認を得て行われる。各参加施設におけるIRB(研究倫理審査委員会:Institutional Review Board)への申請は倫理的に必要としないが、各参加施設の取り決めに従う。

(5) プロトコール内容変更について

本研究のプロトコールの部分的変更は、石川県立中央病院の倫理委員会の取り決めに従って、必要があれば審査承認を得る。プロトコール内容の変更ではなく、文面の解釈上のばらつきを減らす、あるいは注意を喚起する目的で研究関係者に配布するプロトコールの補足説明に関しては、研究事務局への報告を要する。

**７．研究組織**

（１）研究代表者

土山　寿志 石川県立中央病院　消化器内科

（２）研究事務局

林　智之 石川県立中央病院　消化器内科

（３）参加施設ならびに施設代表者(施設名50音順)

| 石川県済生会金沢病院 | 消化器科 | 代田　幸博 |
| --- | --- | --- |
| 石川県立中央病院 | 消化器内科 | 土山　寿志 |
| 金沢医療センター | 消化器科 | 太田　肇 |
| 金沢社会保険病院 | 内科 | 三輪　一博 |
| 金沢市立病院 | 消化器内科 | 辻　宏和 |
| 金沢赤十字病院 | 消化器科 | 寺崎　修一 |
| 小松市民病院 | 消化器内科 | 又野　豊 |
| 珠洲市総合病院 | 内科 | 辻　国広 |
| 北陸病院 | 消化器科 | 増永　高晴 |

**８．結果の報告**

研究成果は、研究代表者が各施設研究者と協議の上で発表責任者を決めて、学会および英文論文に公表する。主たる公表論文は、最終解析終了後に英文誌に投稿する。

なお、本試験では結果の中間解析は行わない。

共著者と記載できる研究者の氏名は、研究代表者、研究事務局及び内視鏡判定に参加した施設の代表者に限る。共著者順は、研究事務局、研究代表者の順は固定し、以後の順番は貢献度または参加意思を表明された順により研究代表者が決定する。

なお、投稿論文の共著者数に制限があり、参加施設の数が人数制限を超える場合は、参加意思表明の順を優先する。残りの施設代表者は、今回の試験において貢献度はまったく同一であるため、Appendixに全内視鏡判定医師として一括して記載することとする。本試験に直接関係のない共同研究者として記載のない研究者名は原則として共著者とはなり得ない。

**９．参考文献**

1. Yao K, Oishi T, Matsui T, Yao T, et al. Novel magnified endoscopic findings of microvascular architecture in intramucosal gastric cancer. Gastrointest Endosc 2002; 56: 279-284.

2. 八尾建史，岩下明徳，八尾恒良，ほか．IIb・IIcと鑑別を要する平坦発赤病変の拡大内視鏡像-微小血管構築所見の良悪性所見における有用性を求めて．胃と腸 2003; 37: 1725-1733，2002.

3. Yao K，Iwashita A，Kikuchi Y，et al．Novel zoom endoscopy technique for visualizing the microvascular architecture in gastric mucosa．Clinical Gastroenterol Hepatol 2005; 3: S23-26.

4. Yao K，Iwashita A，Tanabe H，et al．Novel zoom endoscopy technique for diagnosis of small flat gastric cancer，a prospective，blind study．Clin Gastroenterol Hepatol 2007; 5: 869-78.

5. Yao K, Yao T, Iwashita A. Determining the horizontal extent of early gastric carcinoma: two modern techniques based on differences in the mucosal microvascular architecture and density between carcinoma and non-carcinomatous mucosa. Dig Endosc 2002; 14: 583-87.

6. 八尾建史，頼岡誠，高木靖寛，ほか．胃粘膜の拡大観察：微小血管構築像を指標とした分化型早期胃癌の境界診断. 胃と腸 2003; 38: 1687-1700

7. 八尾建史，松井敏幸，岩下明徳．早期胃癌診断におけるNBI併用拡大内視鏡の臨床応用．日消誌2007; 104: 16-23

8. 八尾建史. 胃拡大内視鏡. 日本メディカルセンター, 東京, 2009; pp1-230

9. Yao K, Anagnostopoulos GK, Ragunath K. Magnifying endoscopy for diagnosing and delineating early gastric cancer. Endoscopy. 2009; 41: 462-7.

10．Ezoe Y, Muto M, Uedo N, et al. Magnifying Narrowband Imaging Versus Conventional White-Light Imaging for Accurate Diagnosis of Gastric Mucosal Cancer. Gastroenterology 2011; 141: 2017-25.

11．伊藤錬磨, 木藤陽介, 中西宏佳,ほか. NBI併用拡大観察診断能のラーニングカーブについての検討. ENDOSCOPIC FORUM FOR DIGESTIVE DISEASE 2012; 28: 76
